# Multispectral label-free *in vivo* cellular imaging of human retinal pigment epithelium using adaptive optics fluorescence lifetime ophthalmoscopy improves feasibility for low emission analysis and increases sensitivity for detecting changes with age and eccentricity

**DOI:** 10.1117/1.JBO.29.S2.S22707

**Published:** 2024-07-03

**Authors:** Karteek Kunala, Janet A. H. Tang, Keith Parkins, Jennifer J. Hunter

**Affiliations:** a Stanford University, Byers Eye Institute, Palo Alto, California, United States; b University of Rochester, Center for Visual Science, Rochester, New York, United States; c University of Rochester, The Institute of Optics, Rochester, New York, United States; d University of Waterloo, School of Optometry and Vision Science, Waterloo, Ontario, Canada

**Keywords:** fluorescence lifetime imaging, *in vivo* imaging, adaptive optics, retinal pigment epithelium, multispectral imaging, lipofuscin and melanin

## Abstract

**Significance:**

Adaptive optics fluorescence lifetime ophthalmoscopy (AOFLIO) provides a label-free approach to observe functional and molecular changes at cellular scale *in vivo*. Adding multispectral capabilities improves interpretation of lifetime fluctuations due to individual fluorophores in the retinal pigment epithelium (RPE).

**Aim:**

To quantify the cellular-scale changes in autofluorescence with age and eccentricity due to variations in lipofuscin, melanin, and melanolipofuscin in RPE using multispectral AOFLIO.

**Approach:**

AOFLIO was performed on six subjects at seven eccentricities. Four imaging channels ($\lambda_{\mathrm{ex}}/\lambda_{\mathrm{em}}$) were used: 473/SSC, 473/LSC, 532/LSC, and 765/NIR. Cells were segmented and the timing signals of each pixel in a cell were combined into a single histogram, which were then used to compute the lifetime and phasor parameters. An ANOVA was performed to investigate eccentricity and spectral effects on each parameter.

**Results:**

A repeatability analysis revealed <11.8% change in lifetime parameters in repeat visits for 532/LSC. The 765/NIR and 532/LSC had eccentricity and age effects similar to previous reports. The 473/LSC had a change in eccentricity with mean lifetime and a phasor component. Both the 473/LSC and 473/SSC had changes in eccentricity in the short lifetime component and its relative contribution. The 473/SSC had no trend in eccentricity in phasor. The comparison across the four channels showed differences in lifetime and phasor parameters.

**Conclusions:**

Multispectral AOFLIO can provide a more comprehensive picture of changes with age and eccentricity. These results indicate that cell segmentation has the potential to allow investigations in low-photon scenarios such as in older or diseased subjects with the co-capture of an NIR channel (such as 765/NIR) with the desired spectral channel. This work represents the first multispectral, cellular-scale fluorescence lifetime comparison *in vivo* in the human RPE and may be a useful method for tracking diseases.

## 1 Introduction

The retinal pigment epithelium (RPE) in the back of the eye sits behind the light-sensitive photoreceptors and is critical for maintaining photoreceptor health and function.^1^ Importantly, the RPE assists photoreceptors to renew the light-absorbing pigment (visual cycle) and digests used photoreceptor outer segments (phagocytosis)^1^. Because the RPE has been implicated in several diseases, such as Stargardt disease,^2^^,^^3^ age-related macular degeneration (AMD),^4^^,^^5^ and retinitis pigmentosa,^6^^,^^7^ understanding and contrasting its compositional and functional changes naturally with age and with disease is vital. A byproduct of the visual cycle brought to the RPE through phagocytosis is lipofuscin. Lipofuscin and melanolipofuscin—autofluorescent lysosomes in the RPE—increase naturally with age, whereas melanin granules reduce with age;^8^ lipofuscin, however, has been shown to accumulate even more rapidly in Stargardt disease^9^ and aggregate in AMD.^10^ However, the role lipofuscin plays in disease progression is still debated; it is not agreed-upon whether it causes toxicity^11^ or plays a passive or even a more complex role in disease progression.^12^^,^^13^ Several studies have investigated the distribution of lipofuscin, melanin, and melanolipofuscin *ex vivo*,^14^^–^^16^ which varies between cells and changes across eccentricity. Before elucidating the roles of lipofuscin and melanin along with other autofluorescent material in disease progression, there is a need to investigate these compositional changes *in vivo* cell-to-cell.

*In vivo* cellular-scale imaging of the RPE mosaic has been made possible by the use of adaptive optics, which corrects the aberrations of the eye, allowing for near diffraction-limited fluorescence imaging of retinal cells.^17^^,^^18^ Many studies rely on structural information where the cellular mosaic is defined by bright borders and dark interiors, likely due to the nucleus blocking autofluorescence from that section of the cell.^18^^–^^21^ A modality is desired that can investigate RPE composition and function beyond structural information. Fluorescence lifetime imaging ophthalmoscopy (FLIO)—which records the time delay from excitation to 1/e of the fluorescence decay—is sensitive to compositional and environmental changes but theoretically independent of fluorescence intensity. FLIO with blue light excitation has been demonstrated in a clinical setting, has shown changes with age and eccentricity,^22^^–^^24^ and has different phenotypes with disease.^25^^–^^27^ Adaptive optics FLIO (AOFLIO), which provides cellular-scale compositional and functional information has been demonstrated *in vivo* in mice,^28^^,^^29^ non-human primates,^30^^,^^31^ and humans.^32^^–^^35^ With the ability to change the excitation wavelength, AOFLIO can probe contributions primarily from lipofuscin, melanin, and melanolipofuscin, AOFLIO has the potential to provide insight into *in vivo* cellular-scale changes with age and eccentricity. Using multiple spectral channels can provide information about different fluorophores and their relative contribution.^36^^,^^37^ The advantage of multispectral fluorescence lifetime imaging is to begin to separate the effects of individual fluorophores in the spectral space.^38^^,^^39^ Multispectral fluorescence lifetime imaging is gaining traction for medical diagnosis^40^^–^^45^ and non-invasive optical biopsy but not often at the cellular-scale or in the human retina. Fluorescence lifetimes are dominated by lipofuscin and melanin in visible (blue or green) and near-infrared (NIR) fluorescence, respectively.^32^^,^^35^^,^^46^^,^^47^ Using these two excitation channels has the potential to be able to separate contributions from different fluorophores. Cellular-scale imaging afforded by AOFLIO is necessary to assess the impact of measured cell-to-cell variations in the concentration of lipofuscin and melanin.^48^ The method of pixel-by-pixel analysis previously used for AOFLIO requires the pooling of histogram data using a kernel in the order of the size of an RPE cell. As a consequence, each pixel in the image has a histogram that is the sum of many surrounding pixels (often a square # by # pixels). Therefore, photons from adjacent cells or regions with overlying blood vessels influence the fluorescence lifetime at each pixel. Although the cell-to-cell variability may be minimal in young healthy eyes, there is a greater variability of sub-cellular features expected in older eyes^8^^,^^49^^,^^50^ that will manifest in a measurable difference in the fluorescence lifetime between adjacent cells. Here, we explore an alternative cell-by-cell method of analysis. By identifying all pixels within a cell and summing to generate a single histogram for each RPE cell, the signal-to noise ratio can be improved without blurring variations between adjacent cells. This methodology may be even more useful in cases of weak fluorescence, such as with blue light excitation, which has a lower maximum permissible exposure compared to longer wavelength light, which can be uncomfortable to subjects with strong photophobia, and for some emission collection bandwidths that have a very weak signal.^32^ The feasibility of combining the pixels from a single cell depends on the visibility and segmentation of the individual cells in the fluorescence intensity data. Using multiple wavelengths for excitation may improve the visibility of the RPE cell mosaic in at least one of the wavelengths. Therefore, we perform a multiwavelength cell segmentation approach as a technique to analyze lifetime data of individual RPE cells.

This study investigates the cell-to-cell fluorescence lifetime fluctuations of the RPE with age and eccentricity in four spectral channels and lays the groundwork for future multispectral longitudinal AOFLIO studies to image older eyes and eyes with disease.

## 2 Methods

### 2.1 Human Subjects

Images from six subjects (3<23 to 28 YO, young; 3>50 to 64 YO, late middle aged) were included in this study. All data was analyzed previously with a pixel-based analysis method; data that was above the photon count threshold (outlined below) was published in previous studies.^32^^,^^34^^,^^35^ Data was collected at the University of Rochester according to the tenets of the Declaration of Helsinki and was approved by the Institutional Review Board. Subjects were screened for risk of complications with pupil dilation and had no history of eye disease or high myopia (<±6 D of refractive error). After obtaining informed consent, subjects were dilated and cyclopleged with 1% tropicamide and 2.5% phenylephrine. IR reflectance, IR fluorescence, blue reflectance, and blue autofluorescence images were taken with a Heidelberg HRA + OCT with a 30° field of view (Heidelberg Engineering, Heidelberg, Germany). OCT scans were taken vertically and horizontally through the locations to be imaged with AOFLIO. These clinical images were used to verify that a subject has a healthy retina. In addition, axial length was measured for each subject prior to AOFLIO using an IOL master. The IR reflectance images were used to navigate around the retina while imaging with AOFLIO. Subject demographics and information are provided in Table 1.

**Table 1:** Subject information.

Subject code	Age	Eye	Sex	Axial length (mm)	Imaging locations	Spectral channel
S1	23	OS	M	25.38	Fovea, 2T, 8T, 12T, 2I, 8I, 12I	765/NIR
					2T, 8T, 12T, 2I, 12I	532/LSC
					2T, 8T, 12T, 2I, 12I	473/LSC
					2T, 8T, 12T, 2I, 12I	473/SSC
S2	26	OD	F	22.59	Fovea, 2T, 8T, 12T, 2I, 8I, 12I	765/NIR
					Fovea, 2T, 8T, 12T, 2I, 8I, 12I	532/LSC
					Fovea, 2T, 8T, 12T, 2I, 8I, 12I	473/LSC
					Fovea, 2T, 8T, 12T, 2I, 8I, 12I	473/SSC
S3	28	OD	F	24.07	2T, 8T, 12T, 2I, 8I, 12I	765/NIR
					Fovea, 2T, 8T, 12T, 2I, 8I, 12I	532/LSC
					Fovea, 8T, 12T, 2I, 8I, 12I	473/LSC
					2T, 8T, 12T, 2I, 8I, 12I	473/SSC
S4	50	OD	M	23.76	2T, 12T, 8I, 12I	765/NIR
					Fovea, 2T, 12T, 8I, 12I	532/LSC
S5	54	OD	F	23.66	Fovea, 12T	765/NIR
					Fovea, 12T	532/LSC
S6	64	OD	F	23.21	8T	765/NIR
					8T	532/LSC

### 2.2 Adaptive optics Fluorescence Lifetime Imaging Ophthalmoscope

Subjects were imaged in the AOFLIO device at a 1.4 deg square field of view. Seven eccentricities were imaged: the fovea, 2 deg, 8 deg, and 12 deg inferior and temporal. AOFLIO has been described previously.^32^ In short, the subject’s aberrations were measured using a Shack-Hartmann wavefront sensor with a 927 nm (φ<20  μW; IPSDS0901C, Inphenix, Livermore, CA) or 847 nm (φ<20  μW; QFLD-850-20S-PM, QPhotonics LLC, Ann Arbor, Michigan, United States) source. The aberrations were corrected in a closed loop using a deformable mirror (DM-97, ALPAO, Montbonnot-Saint-Martin, FR). An NIR reflectance channel was used to navigate across the retina. The fluorescence was excited using a supercontinuum laser (SuperK EXTREME FIU-15, NKT Photonics, Birkerød, DK) filtered to the desired wavelength with a tunable filter (SuperK VARIA, NKT Photononics). The source has an 80 MHz repetition frequency and a 50 ps pulse width. Four imaging paradigms were used that are referred to in this paper using the format excitation wavelength/emission collection band: 765/NIR [765Δ30  nm/long spectral channel (800 to 900 nm)], 532/LSC [532Δ10 nm/long spectral channel (575 to 725 nm)], 473/LSC [473Δ10 nm/long spectral channel (575 to 725 nm)], and 473/SSC [473Δ10  nm/short spectral channel (500 to 550 nm)]. The channels were selected to acquire fluorescence from the three main autofluorescence organelles in the RPE arising from lipofuscin, melanin, and melanolipofuscin, where 473 and 532 nm excite lipofuscin and melanolipofuscin and 765 nm excites primarily melanin and melanolipofuscin. Two 473 nm channels were used to be consistent with current clinical FLIO devices. The 532 nm excitation was chosen to avoid the macular pigment absorption, improving signal at the fovea, as compared to excitation at 473 nm.^51^^,^^52^ The combination of data from all four channels should give a better understanding of various fluorophores in RPE. For the short-wavelength autofluorescence channels (SWAF; excitation with 532 or 473 nm), a 796 nm reflectance source (φ<136.0  μW, S790-G-I-15, Cork, Ireland) was used for navigation. And for the near-infrared wavelength autofluorescence (NIRAF, 765/NIR channel), a single source was used for excitation and navigation. The excitation, collection, and length of exposure for each channel are shown in Table 2. Because the 473/LSC and 532/LSC were correlated^47^ and for patient comfort, the late middle-aged subjects were not imaged with the 473/SSC and 473/LSC. For those that were, all four channels were collected within 3 months to minimize variations due to biological changes. SWAF channels were collected more than 24 h apart for light safety. The 473/LSC and 473/SSC imitate clinical FLIO.^53^

**Table 2:** Multiwavelength fluorescence spectral channels in AOFLIO.

Channel	Excitation	Emission	Imaging duration	Maximum power (μW)	Confocal pinhole size
765/NIR	765Δ30 nm	800 to 900 nm	120 s	300	2.7 ADD
532/LSC	532Δ10 nm	575 to 725 nm	20 to 30 s	21	2.3 ADD
473/LSC	473Δ10 nm	575 to 725 nm	30 s	20	2.9 ADD
473/SSC	473Δ10 nm	500 to 550 nm	30 s	20	2.9 ADD

ADD = Airy disk diameter.

Fluorescence lifetime measurements for each excitation and emission combination were made on separate study visits. The emitted fluorescence was collected using a hybrid photomultiplier tube (PMT) (532/LSC, 473/LSC, and 473/SSC: HPM-100-40; 765/NIR: HPM-100-50C, TE cooled, Becker & Hickl, Berlin, Germany) through a confocal pinhole with size dependent on the wavelength of interest (Table 2). The 796 nm reflectance was collected using H7422-50 PMT (Hamamatsu Corporation, Bridgewater, New Jersey, United States) through a 1.1 ADD confocal pinhole. Depending on the channel of interest the light reflected from the eye deviated to the specific PMT using dichroic and filters to split the channels where 750DCXXR dichroic (Chroma Technology Corp., Bellows Falls, Vermont, United States) was used to split the SWAF with the NIR collection and the FF875/Di01 dichroic (Semrock, Rochester, New York, United States) was used to split the NIR collection with the wavefront sensing. The NIR reflectance and the NIRAF was split using T800lpxr-xt-UF2 (Chroma Technology Corp., Bellows Falls, Vermont, United States). The 532/LSC and 473/LSC were captured through a FF01-650/150 bandpass filter (Semrock, Rochester, New York, United States), whereas 473/SSC was captured through a FF03-525/50 bandpass filter (Semrock, Rochester, New York, United States). NIRAF was collected using four filters (CT853/93 × 2 and CT850/100 × 2 (Chroma Technology Corp., Bellows Falls, Vermont, United States)) to remove any potential excitation light bleed through. The lifetime data were sent to an electronic splitter (HPMCON-02, Becker & Hickl) to allow for real-time readout to the adaptive optics scanning light ophthalmoscope (AOSLO) software, which enabled two important capabilities. First, the splitter allowed for optimizing the location of the confocal pinhole to account for the subject’s individual longitudinal chromatic aberration;^18^ the optimization was done after moving to each new location. Second, it allowed for the fluorescence intensity to be co-registered with the photoreceptor reflectance image. For the SWAF channels, this was done in sections to allow for correction of the transverse chromatic aberration as described here.^19^ The timing data of the fluorescence were recorded using a time-correlated single-photon counter that records the time from the excitation pulse to when a photon is received; this is aggregated over many excitation pulses to generate a histogram for each pixel, and the image acquisition length for each channel is shown in Table 2. The reflectance intensity, fluorescence intensity,^54^ and fluorescence lifetime data were desinusoided and strip registered with custom software;^55^^,^^56^ the intensity of the fluorescence lifetime signal was compared with the fluorescence intensity images from the electronic splitter to ensure accuracy.

The borders of the visible RPE cells in the fluorescence intensity images were outlined by hand with a single-pixel width in Photoshop (Adobe, San Jose, California, United States) to create a mask, similar to previously described.^18^^,^^19^ A different grader corrected the segmentation. Intensity images with RPE cell visibility occluded by noise were put through a low-pass filter to aid in cell segmentation and to create a mask. Each mask was subsequently applied to unfiltered histogram data for lifetime analysis. Hence, AOFLIO data were not impacted by this low-pass filter. Cells partially outside the field of view were excluded. All channels collected in each location were overlapped; the channel with the best cell visibility was used to segment the RPE cells—either the 532/LSC or the 765/NIR. The same mask was applied to all four channels. The cell size (area) in pixels was computed and converted to micrometers using the axial length data from each subject. All of the photons within an encircled cell were combined into a single histogram; the cell borders were excluded to avoid duplicating the pixels. A double-exponential fit (SPCImage v8.3, Becker and Hickl, Berlin, Delaware, United States) and phasor analysis (which spatially separates the data by analyzing in the Fourier space^57^ conducted in custom MATLAB software^30^) were performed on the histogram. The double exponential fit was preferred instead of a triple to be consistent with the pixel-wise approach^32^ we published where the relative contribution of the triple exponential was very small (<5%). The double-exponential fit was executed using the maximum likelihood estimation algorithm with 2 components and a minimum threshold of 300 photons per histogram.^58^ The weighted mean lifetime was also computed ($\tau_{m}=a_{1}\tau_{1}+a_{2}\tau_{2}$ where $a_{1}+a_{2}=1$) where $\tau_{1},\tau_{2}$ are the individual lifetime components and $a_{1}$, $a_{2}$ are their respective weights. The phasor parameters (g,s) were computed as the real and imaginary components, respectively, of the Fourier transform of the raw histograms, evaluated at the repetition frequency of the laser. To test the sensitivity of the segmentation, a single image was analyzed by moving the mask by 1 pixel, by 15 pixels, and by rotating the segmentation 90 deg. The lifetime data from each of those three scenarios were compared to the lifetime data of the original segmentation using intraclass correlation (ICC) (two-way mixed effects, consistency model).

The repeatability of segmentation was tested in three separate scenarios using ICC (two-way mixed effects, absolute agreement model) and the absolute value of the differences. The first of the three scenarios compared the forward scan and backward scan (clockwise and counterclockwise motion of the resonant scanner) for 3 locations (687 cells), where there should be no differences in signal. The second scenario compared the variation in each cell across 2 visits where there was cell visibility in both visits. Rotation between visits was not accounted for. The last scenario tested the repeatability of averaging the lifetime and phasor data across each image after cell segmentation analysis. Eleven locations in three subjects across multiple visits were compared; these images may or may not have cell visibility in both visits; rotation between visits was also not accounted for. Frame-averaging was used for most of the data analysis/statistics for eccentricity, age, and wavelength comparison in this manuscript. ICC values less than 0.5, 0.5 to 0.75, 0.75 to 0.9, and greater than 0.9 were considered to have poor, moderate, good, and excellent agreement, respectively.^59^

To compare the cell-encircling approach to the traditional pixel-wise method, the cell-encircled data was compared to data analyzed using the pixel-wise method previously described.^32^^,^^35^ This was done in 4 subjects with 18 total locations in the 532/LSC. Comparisons were made using ICC (two-way mixed effects, consistency model) and a repeated-measures ANOVA.

In SPSS (IBM, Armonk, New York, United States) to explore the data’s dependence on eccentricity with each of the four imaging paradigms, ANOVAs were performed on the lifetime exponential parameters and the phasor parameters across all images with eccentricity as a fixed factor. No statistical test was performed across age due to the low amount of data captured with older normals. To compare the four spectral channels, a repeated-measures ANOVA on the exponential and phasor parameters was computed with eccentricity as a fixed factor. P-values of 0.05 or less were considered significant. To understand effects found in the ANOVA, unpaired Student’s t-tests and the linear trendline across the data points were computed in Excel (Microsoft, Redmond, Washington, United States). The Holm-Bonferroni correction was applied to the Student’s t-tests to reduce the possibility of a type I error.^60^

## 3 Results

### 3.1 Comparison to the Pixel-Based Method

**Fig. 1:**
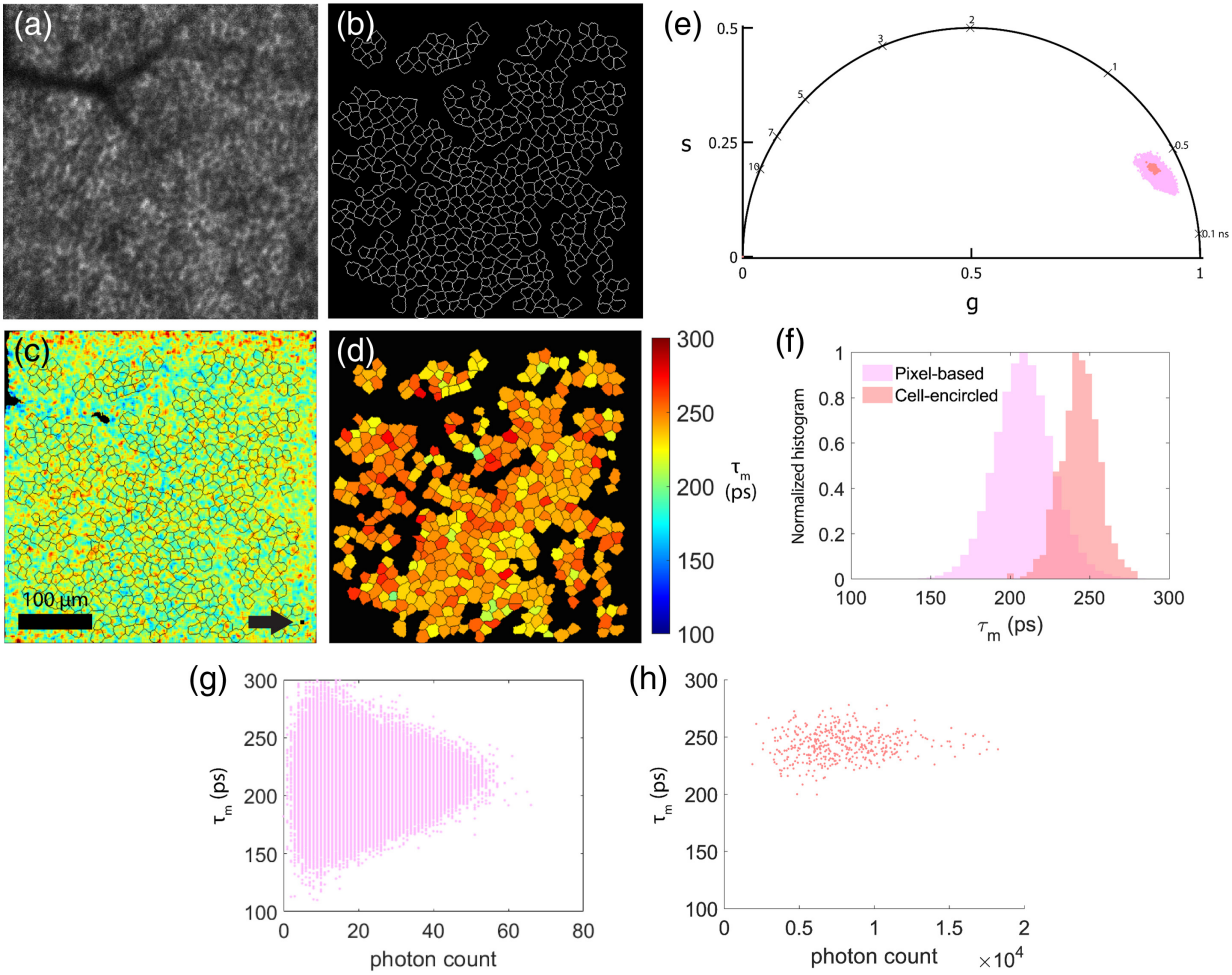
An example of segmentation (b) of the fluorescence intensity image (a) in a 23 YO male at 12°T collected using the 532/LSC where all of the histograms within a cell are combined, and an exponential fit performed to give a single mean fluorescence lifetime in a single cell (d) compared to the mean fluorescence lifetime processed with a kernel of 7 pixels across the image and overlaid with cell borders for visual guide (c); the square shows the kernel size of 7 pixels (c). The phasor has a similar mean but the standard deviation is reduced with segmentation (e). (f) The normalized histogram of the cell-encircled and pixel-based lifetimes. (g) and (h) $\tau_{m}$ versus photon count contour plots for the unbinned pixel-wise and cell-encircled analyses, respectively.

Twenty-nine images were segmented across six subjects (23 to 64 YO) at 1-7 eccentricities in each subject (Table 1). An example of the fluorescence intensity and segmentation captured with 532/LSC is shown in Figs. 1(a) and 1(b). The segmented mean fluorescence lifetime is shown in Fig. 1(d) and the pixel-based analysis with the overlayed cell segmentation (for visual aid) is shown in Fig. 1(c).

The mean of the segmented fluorescence lifetime is longer compared to the pixel-based analysis [Fig. 1(f)]. Each data point in the cell-encircled histogram and phasor is a cell; in the pixel-based analysis, each data point represents a kernel of 7 pixels. The $\tau_{m}$ value is not dependent on the photon count [Figs. 1(g) and 1(h)]. The phasor is slightly shifted between analyses, but the major difference is the much smaller standard deviation [Fig. 1(e)]. $\tau_{m}$ was consistently longer in the segmented images; the mean of each of the parameters between the two types of analyses had good to excellent agreement (Table 3). The average cell size across all segmented images is $219.7\pm 73.5\;{\mu m}^{2}$ with a range of 126.6 to $398.9\;{\mu m}^{2}$. This is similar to the RPE cell sizes reported by Granger et al.^19^

**Table 3:** Comparison of cell-encircled with pixel-based analysis and the intraclass correlation between each lifetime and phasor parameter.

	$\tau_{m}$	$\tau_{1}$	$\tau_{2}$	$a_{1}$	g	s
Repeated measures ANOVA (p-value)	**<0.001**	**<0.001**	**<0.001**	**<0.001**	**<0.001**	**<0.001**
ICC	0.881	0.768	0.859	0.836	0.963	0.922

Note: Significant values are bolded.

**Fig. 2:**
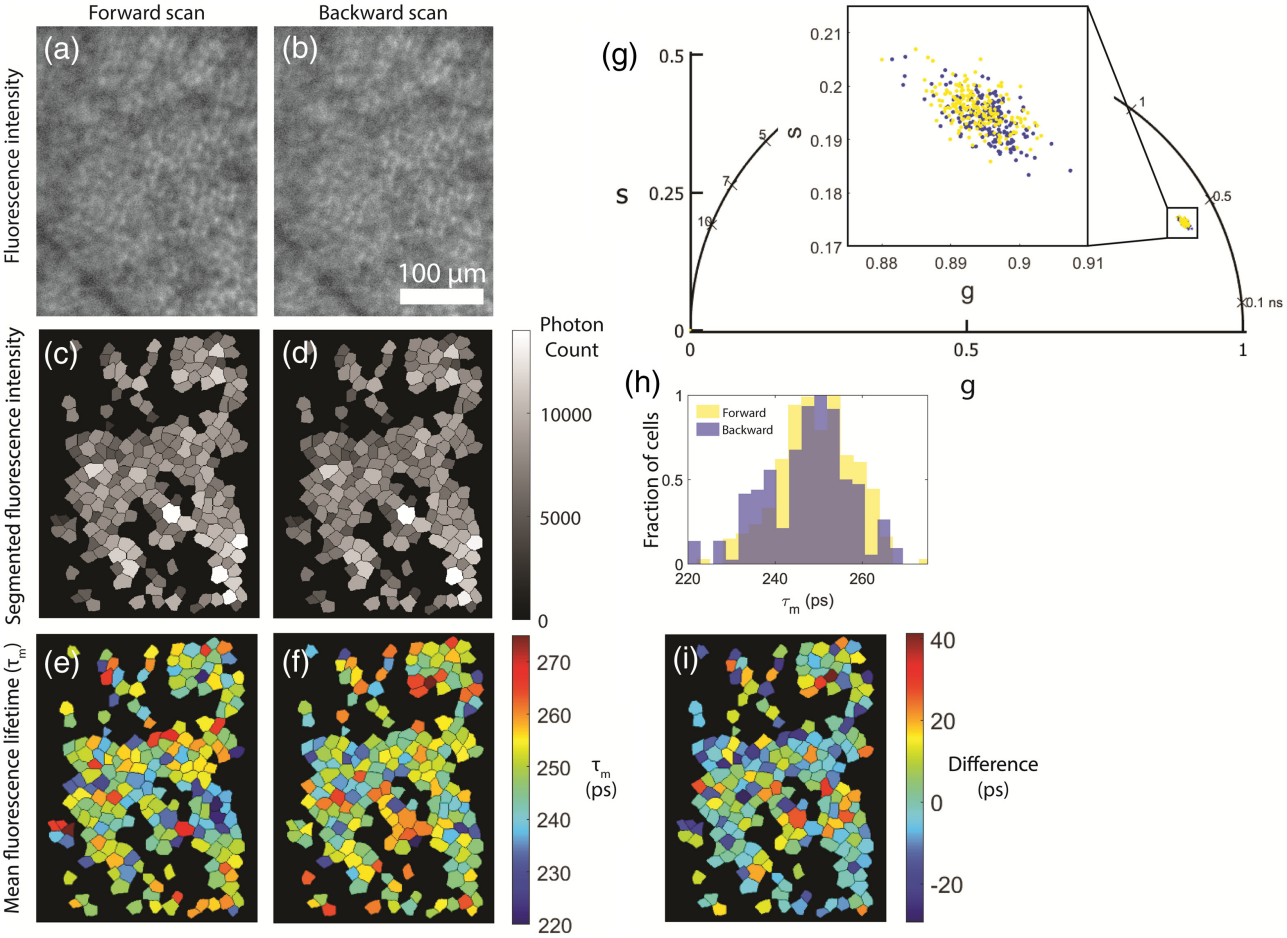
A comparison of the forward and backward scans of a single acquisition in a 28 YO female at 12°T. (a) and (b) The fluorescence intensity of the forward and backward scan, respectively. (c) and (d) The segmented fluorescence intensity and (e) and (f) the $\tau_{m}$ values. (g) The phasor for the forward and backward scan. (h) The histogram of $\tau_{m}$ and (i) the difference of the forward minus the backward scan.

To investigate the sensitivity on small misalignments, one image was processed with a mask shifted as shown in Table 4. Shifting the segmentation mask by 1 pixel had excellent agreement, shifting it by 15 pixels had poor to moderate agreement, and rotating the mask by 90 deg had no agreement with the original mask (Table 4). Across the forward and backward scan, the phasor parameters had good to excellent agreement while the exponential fit parameters had poor to moderate agreement. The absolute value of the differences showed a difference in $\tau_{m}$ of 16.7±14.4  ps (Table 5). Figure 2 shows an example of the forward and backward scans for a single location. A cell-to-cell test-retest comparison across two visits revealed excellent repeatability for the phasor parameters, moderate for $\tau_{m}$, and poor to no repeatability for $\tau_{2}$, $a_{1}$, and $\tau_{1}$, and the average absolute value of the differences was small (Table 5).

**Table 4:** Intraclass correlation coefficient comparing movements of the segmentation mask in one location.

Case	$\tau_{m}$	$\tau_{1}$	$\tau_{2}$	$a_{1}$	g	s
1 pixel shift	0.979	0.976	0.977	0.976	0.980	0.986
15 pixels shift	0.517	0.411	0.366	0.418	0.573	0.704
90 deg rotation	ns	ns	ns	ns	ns	ns

Note: ns is not significant.

**Table 5:** Cell-to-cell repeatability analysis.

	$\tau_{m}$	$\tau_{1}$	$\tau_{2}$	$a_{1}$	g	s
Forward and backward scan (n=3 subjects, 3 locations, 687 cells)						
ICC	0.698	0.228	0.378	0.360	0.930	0.956
|Differences|	16.7 ± 14.4	18.1 ± 15.6	90.5 ± 76.8	1.78 ± 1.46	0.006 ± 0.006	0.005 ± 0.004
% Difference	6.75 ± 5.83%	11.8 ± 10.2%	9.85 ± 8.35%	2.04 ± 1.67%	0.68 ± 0.66%	2.65 ± 2.16%
Test-retest across two visits (n=3 subjects, 3 locations, 1629 cells)						
ICC	0.726	ns	0.378	0.373	0.946	0.949
|Differences|	15.8 ± 12.8	17.4 ± 14.1	78.8 ± 66.9	1.56 ± 1.39	0.005 ± 0.004	0.006 ± 0.004
% Difference	6.31 ± 5.13%	11.3 ± 9.07%	8.43 ± 7.16%	1.79 ± 1.58%	0.60 ± 0.48%	2.86 ± 2.07%

Note: ns is not significant.

To understand the repeatability of averaged data with the cell-encircling method, a test-retest analysis was additionally conducted using the average and standard deviation across a location with the results shown in Table 6. The average and standard deviation of the exponential fit and phasor parameters across 11 locations taken 14 to 77 days apart had good to excellent repeatability (Table 6) with small differences between the two visits.

**Table 6:** Average across a location - repeatability analysis.

	$\tau_{m}$	$\tau_{1}$	$\tau_{2}$	$a_{1}$	g	s
Average and standard deviation across an image (n=3 subjects, 11 locations total)						
ICC	0.958	0.842	0.950	0.948	0.986	0.914
|Differences|	9.4 ± 5.5	8.3 ± 3.9	22.3 ± 18.1	0.40 ± 0.37	0.003 ± 0.002	0.008 ± 0.008
% Difference	3.83 ± 2.25%	5.50 ± 2.61%	2.40 ± 1.95%	0.46 ± 0.42%	0.38 ± 0.23%	4.04 ± 3.83%

### 3.2 Multispectral Analysis

#### 3.2.1 765/NIR channel

**Fig. 3:**
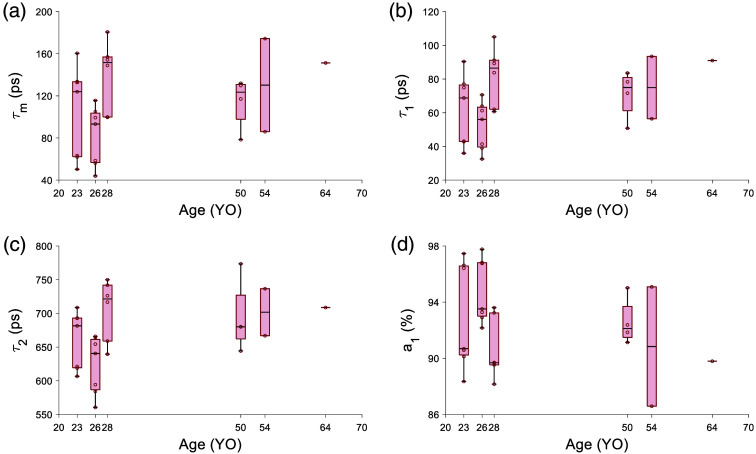
Fluorescence lifetime parameters change with age in the 765/NIR channel. (a) $\tau_{m}$, (b) $\tau_{1}$ and (c) $\tau_{2}$ tending toward a longer lifetime with age. $a_{1}$ (d) tends toward a shorter lifetime with age.

**Fig. 4:**
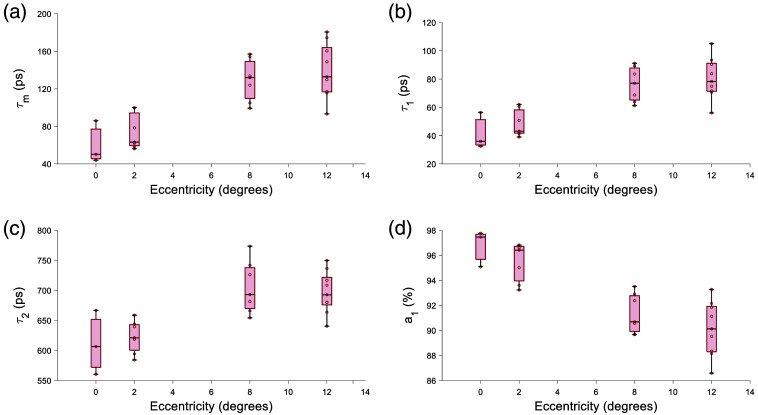
Lifetime parameters change with eccentricity in the 765/NIR channel. A clear increase in (a) $\tau_{m}$, (b) $\tau_{1}$, and (c) $\tau_{2}$ and a decrease in panel (d) $a_{1}$ was observed. All were statistically significant with eccentricity with p<0.001 for all parameters.

**Fig. 5:**
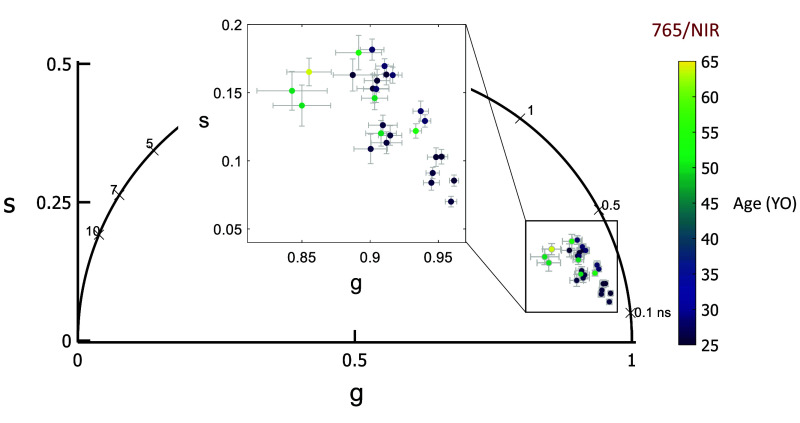
Phasor location moves up and to the left toward longer lifetimes with age in the 765/NIR channel.

The results from an ANOVA of the mean lifetime and phasor parameter across each location are tabulated in Table 7 for 765/NIR channel. With age, $\tau_{m}$, $\tau_{1}$, and $\tau_{2}$ [Figs. 3(a)–3(c)] tended toward longer lifetimes and $a_{1}$ seemed to decrease, though not monotonically [Fig. 3(d)]. The effects may be due to inter-subject variability and need to be tested with more subjects. With eccentricity, there was a clear increase in $\tau_{m}$, $\tau_{1}$, and $\tau_{2}$ and a decrease in $a_{1}$ (Fig. 4). The phasor location moves toward longer lifetime with age (Fig. 5) and eccentricity (Fig. S1 in the Supplementary Material). The eccentricity effect is more easily recognizable, similar to results in previous studies.^34^

**Table 7:** p-values from the ANOVA within 765/NIR channel.

Parameter	$\tau_{m}$	$\tau_{1}$	$\tau_{2}$	$a_{1}$	g	s
Eccentricity	**<0.001**	**<0.001**	**<0.001**	**<0.001**	**<0.001**	**<0.001**

Note: Significant values are bolded.

#### 3.2.2 532/LSC channel

**Fig. 6:**
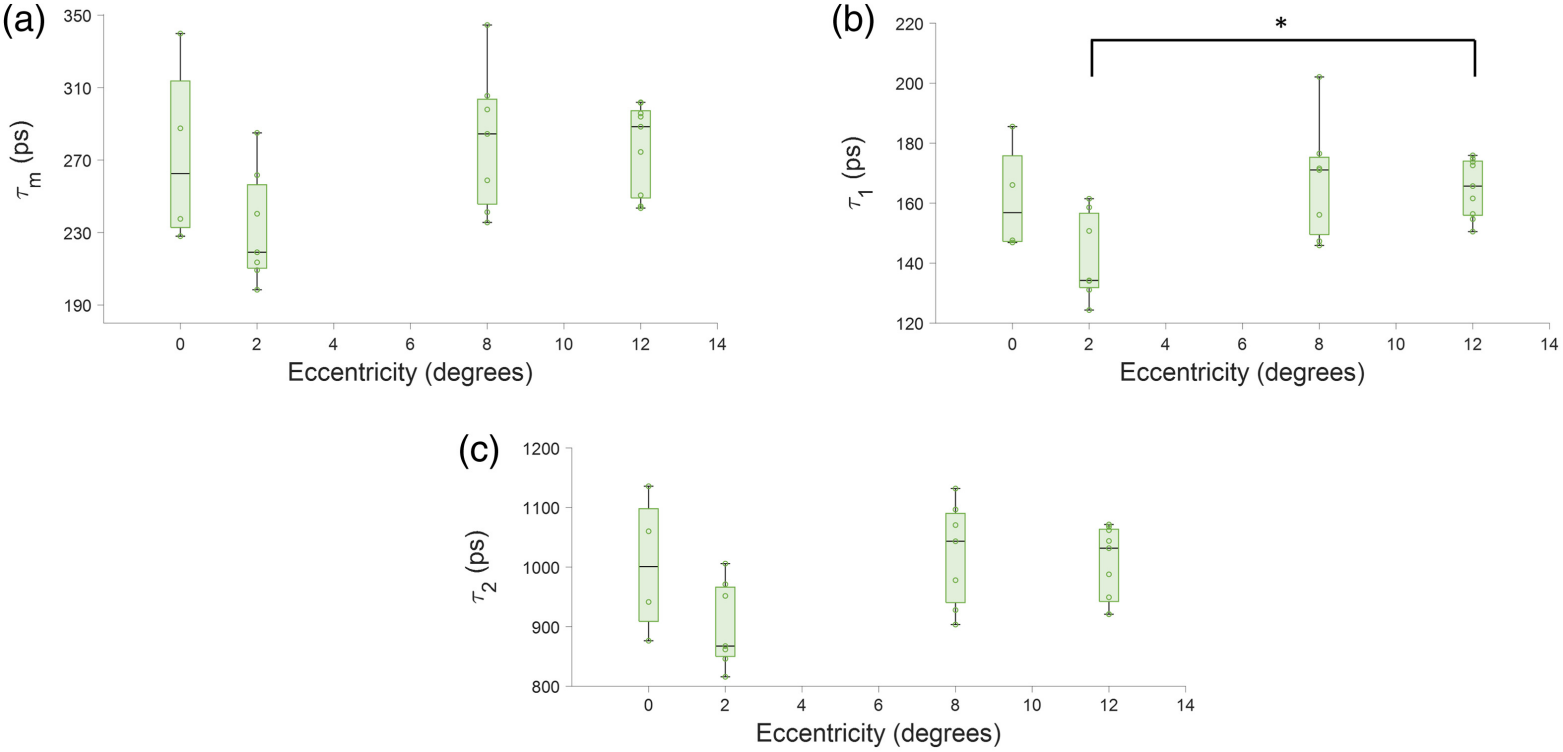
Fluorescence lifetime parameters with eccentricity in the 532/LSC channel. (a) $\tau_{m}$, (b) $\tau_{1}$, and (c) $\tau_{2}$. * denotes a statistical difference with a corrected Student’s t-test.

**Fig. 7:**
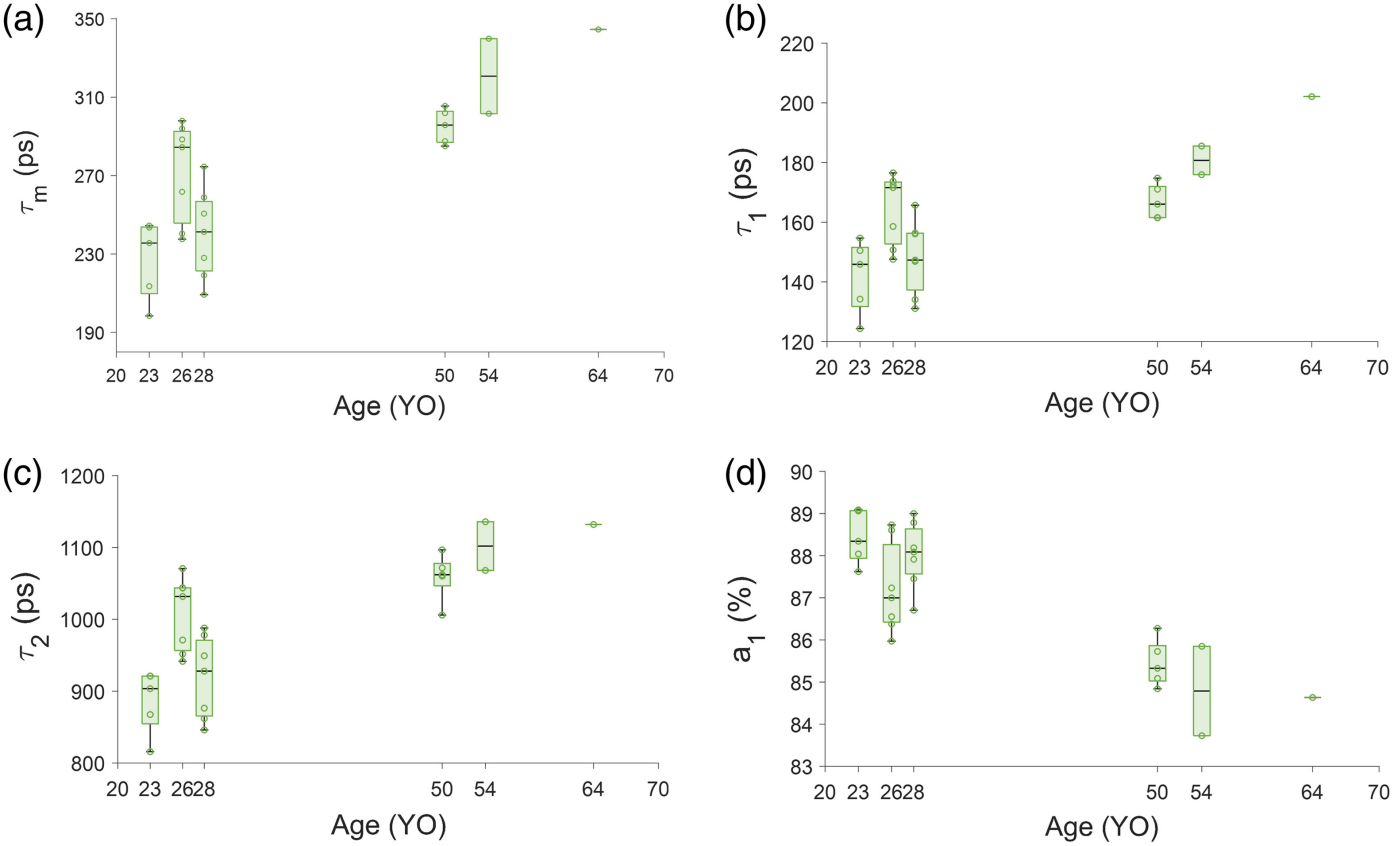
Fluorescence lifetime parameters with age in the 532/LSC channel. (a) $\tau_{m}$, (b) $\tau_{1}$, and (c) $\tau_{2}$ shows the clear increase in lifetime with age. (d) The $a_{1}$ reduce with age, which has a similar effect of increasing the mean lifetime with age.

The results from an ANOVA of the mean lifetime and phasor parameters across each location are tabulated in Table 8 for 532/LSC channel. The eccentricity effect detected in $\tau_{1}$ and $\tau_{2}$ is unclear (Fig. 6) with no particular trend of the means. A corrected t-test between each eccentricity found 2 deg to be different from 12 deg only for $\tau_{1}$ (p-value=0.005, corrected α=0.008). The phasor location moved up and to the left with eccentricity (Fig. S2 in the Supplementary Material) and age (Fig. S3 in the Supplementary Material), similar to what we observed with the previous pixel-based analysis.^47^ In the 532/LSC with age, there was a clear increase in $\tau_{m}$, $\tau_{1}$, and $\tau_{2}$ [Figs. 7(a)–7(c)] and a decrease in $a_{1}$ [Fig. 7(d)].

**Table 8:** p-values from the ANOVA within 532/LSC channel.

Parameter	$\tau_{m}$	$\tau_{1}$	$\tau_{2}$	$a_{1}$	g	s
Eccentricity	0.053	**0.019**	**0.042**	0.250	0.224	0.185

Note: Significant values are bolded.

#### 3.2.3 473/LSC and 473/SSC channel

**Fig. 8:**
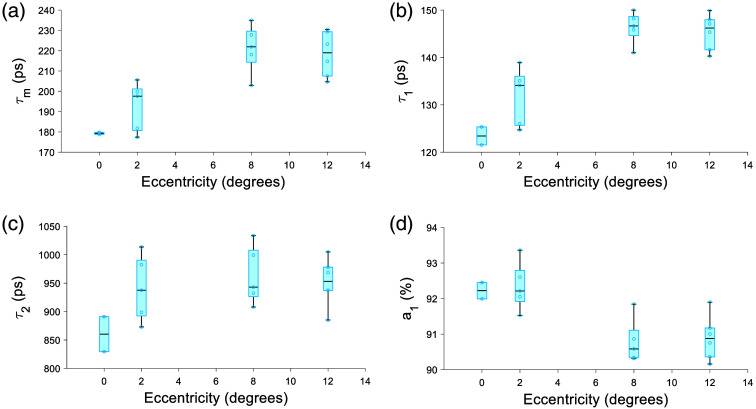
Fluorescence lifetime parameters in 473/LSC channel changes with eccentricity. A clear increase in (a) $\tau_{m}$, (b) $\tau_{1}$ and decrease in panel (d) $a_{1}$ can be seen while (c) $\tau_{2}$ tends toward a longer lifetime, but the effect is weak.

The 473/LSC and 473/SSC did not have any data from the late middle-aged population due to low collected photon counts that did not meet our threshold criteria of 300 photons per Histogram. Therefore, an age effect was not investigated. The 473/LSC has an eccentricity effect in $\tau_{m}$, $\tau_{1}$, $a_{1}$, and s (Table 9). $\tau_{m}$, $\tau_{1}$, and $\tau_{2}$ increase with eccentricity, though not monotonically [Figs. 8(a)–8(c)]. $a_{1}$ decreases with eccentricity [Fig. 8(d)]. The phasor location for 473/LSC moves toward longer lifetime with eccentricity (Fig. S4 in the Supplementary Material), similar to the 532/LSC (Fig. S3 in the Supplementary Material).

**Table 9:** Eccentricity effects within 473/LSC and 473/SSC channels (p-values from the ANOVA).

Channel	$\tau_{m}$	$\tau_{1}$	$\tau_{2}$	$a_{1}$	g	s
473/LSC	**<0.001**	**<0.001**	0.136	**0.003**	0.314	**0.003**
473/SSC	0.099	**0.007**	0.121	**0.020**	0.228	0.426

Note: Significant values are bolded.

**Fig. 9:**
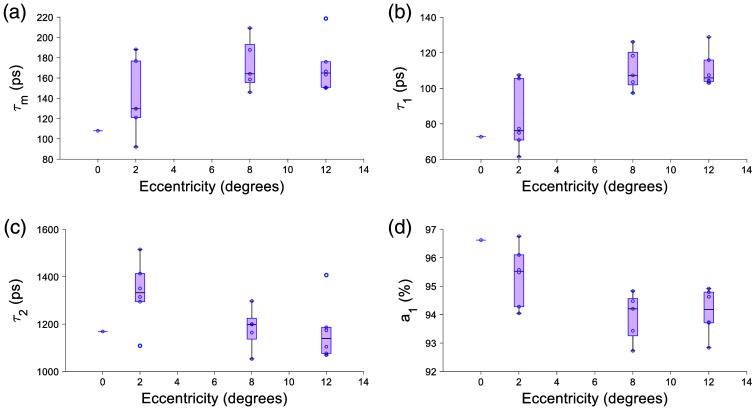
Fluorescence lifetime parameters in 473/SSC channel with eccentricity. (a) $\tau_{m}$ tends toward longer lifetime and (b) $\tau_{1}$ shows a clear increase in lifetime with eccentricity. (c) $\tau_{2}$ does not a particular trend. And a decrease in panel (d) $a_{1}$ can be seen with eccentricity.

**Fig. 10:**
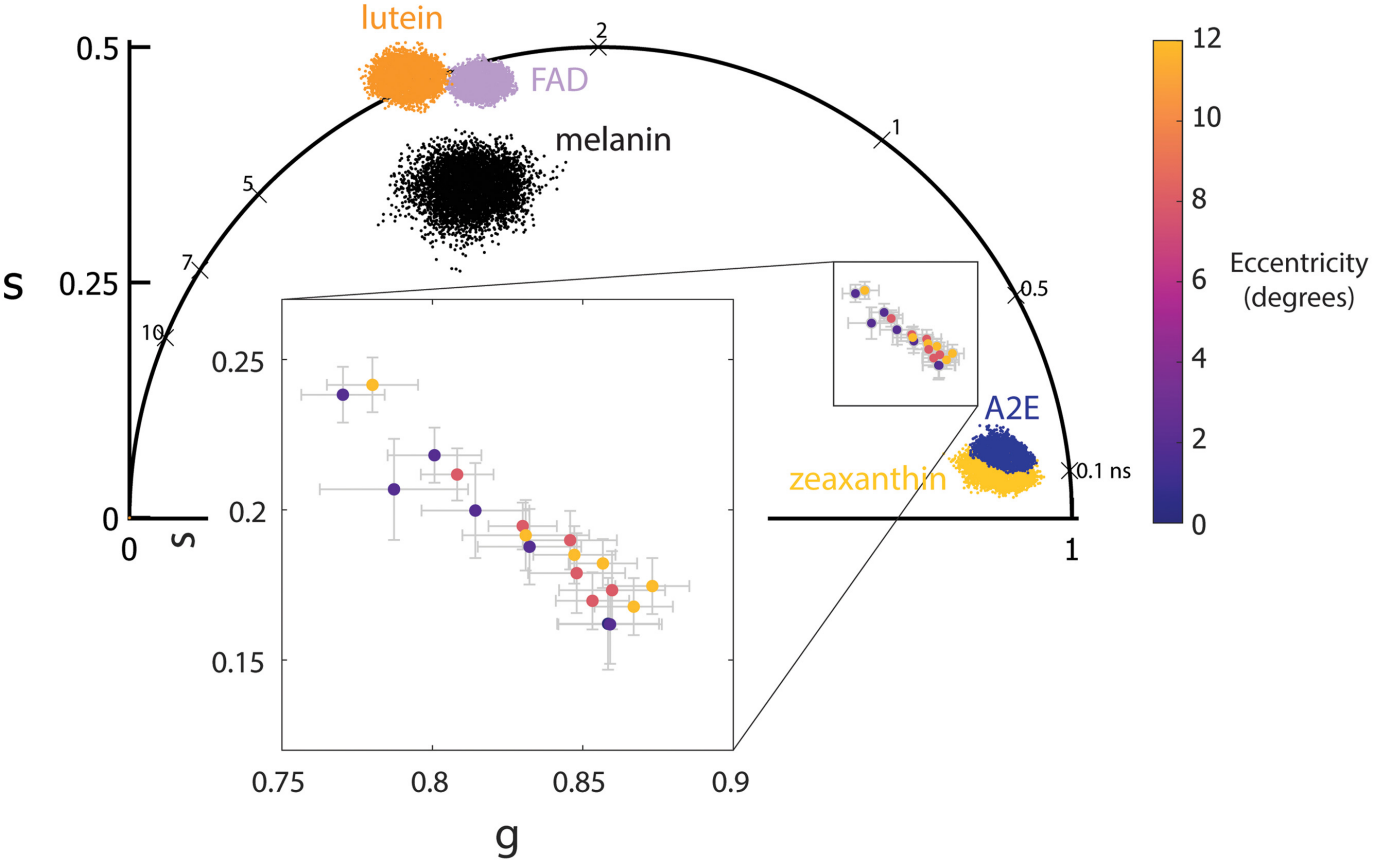
In 473/SSC channel, no eccentricity effect was seen in the phasor plot. The phasor fingerprint from our AOFLIO system (taken from Tang et al.^47^) is also shown for comparison with the in vivo cell analysis data.

The 473/SSC had an increase in $\tau_{m}$ and $\tau_{1}$ with eccentricity [Figs. 9(a) and 9(b)]; $a_{1}$ decreases with eccentricity [Fig. 9(d)]. The phasor result in the 473/SSC was not significant and had no clear trend (Fig. 10).

#### 3.2.4 Multispectral comparison

**Fig. 11:**
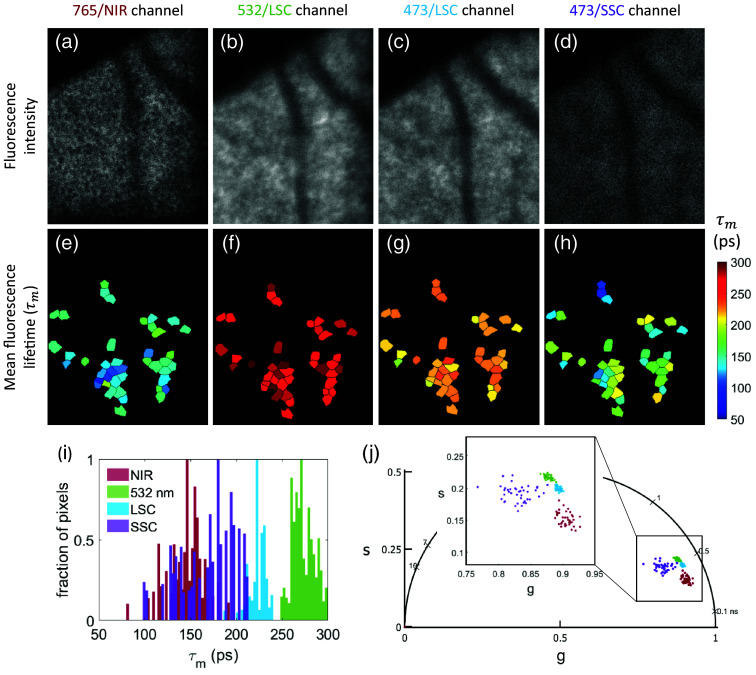
A comparison across the four spectral channels in a 28 YO female at 12°I (S3). (a)–(d) The fluorescence intensity for the 765/NIR, 532/LSC, 473/LSC, and 473/SSC, respectively. (e)–(h) The corresponding fluorescence lifetime. (i) The histogram of the $\tau_{m}$ values for each channel and (j) is the phasor data for each spectral channel.

**Fig. 12:**
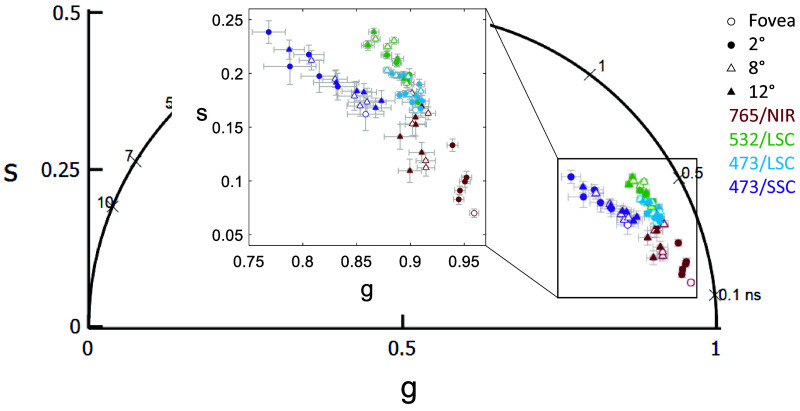
A comparison of phasor coordinates across the four spectral channels reveals the difference with eccentricity and wavelength. The phasor data shifted up and to the left with eccentricity in 765/NIR, 532/LSC and 473/LSC while 473/SSC did not show any clear trend. The 532/LSC and the 473/LSC lie along the same line just shifted showing the correlation between the two channels.

A repeated-measures ANOVA across all four channels revealed differences in all parameters with only $a_{1}$ having an eccentricity effect. The results are tabulated in Table 10. An example image of the same location with fluorescence intensity well overlapped in all four channels is shown in Figs. 11 [Figs. 11(a)–11(d)]. $\tau_{m}$ is shorter in the 473/SSC and longest in the 532/LSC [Figs. 11(f)–11(h)] among the short wavelength channels and the 765/NIR has the shortest $\tau_{m}$ among the four wavelengths. In this example, each channel has a different phasor location and standard deviation [Fig. 11(j)]. The phasor location across the four wavelengths for all the data collected is shown in Fig. 12 with eccentricity as a variable. The phasor location between the 473/LSC and 532/LSC shifts along a line with the same slope, but the 473/SSC has a different location and slope. The 765/NIR has a cluster separated from the three SWAF channels.

**Table 10:** Repeated Measures ANOVA across four spectral channels (765/NIR, 532/LSC, 473/LSC, and 473/SSC).

p-value	$\tau_{m}$	$\tau_{1}$	$\tau_{2}$	$a_{1}$	g	s
Between channels	**<0.001**	**<0.001**	**<0.001**	**<0.001**	**<0.001**	**<0.001**
Eccentricity interaction between channels	0.378	0.337	0.324	**0.013**	0.061	0.330

Note: Significant values are bolded.

## 4 Discussion

This study explores the cell-to-cell fluorescence lifetimes of the RPE with age and eccentricity in four spectral channels. The feasibility of the cell encircling method was explored by encouraging repeatability and insensitivity to small shifts. It has the potential to be useful for multispectral investigations, with the limitation of having the emission spectrum of each fluorophore at different wavelengths and for low-light situations, such as the AOFLIO 473/SSC. The cell sizes fit in a normative range, demonstrating the validity of the segmentation.

The cell-encircled analysis, as compared to the pixel-wise analysis, had longer lifetimes in the exponential-fit parameters and showed a significant difference in the phasor parameters; it also had more photons per histogram. Those differences are not due to a better multi-exponential fit, as all included pixels and cells were above a photon-count threshold to prevent inaccurate fits^58^ and the reduced chi-square ($\chi^{2}$) fitness parameter value for each analysis was close to 1.0 (cell-encircled: 1.12±0.11; pixel-wise: 1.04±0.04). An alternate explanation considers that combining all the histograms in a cell into a single decay curve increases the number of photons included for exponential decay curve fitting, which could have a longer tail resulting in longer lifetime. Interestingly, this photon count effect is only observed when the photon count difference is very large (between analysis methods) and not when the change is small, such as across a single image [Figs. 1(g) and 1(h)].

The good to excellent correlation with a 1-pixel shift and poor to good correlation with a 15-pixel shift indicate that this analysis is not sensitive to small misalignment but does provide a unique comparison of adjacent cells. Insensitivity to small misalignments is critical as one channel with cellular visibility (here: 765/NIR or 532/LSC) was used to segment the other channels. The 473/LSC and 473/SSC rarely or never had cell visibility. This analysis was only possible by aligning these channels to the segmented channel using similar fluorescence patterns and blood vessel locations and occasionally with the help of photoreceptor reflectance images from each channel. Therefore, it is reassuring that misalignment by a few pixels will not alter the results and conclusions.

The standard deviation of the phasor parameters is smaller in the cell-encircled compared to the pixel-based analysis. Using a best-fit line of g and s measured in 532/LSC channel with age, the difference predicted between subjects a decade apart was 0.013 in both g and s. To detect this difference with 80% power (the probability of avoiding a Type II error), the pixel-wise analysis with an average standard deviation of (g: 0.019, s: 0.017) would require 27 (g: 27, s: 25) participants in each group. Cell-encircling analysis with an average standard deviation of (g: 0.005, s: 0.004) would require 4 (g: 4, s: 1) subjects in each group.^61^^,^^62^ A similar effect was observed in the NIR/765 channel where the average standard deviation of (g: 0.0097, s: 0.0081) in cell-encircling would require 22 (g: 10, s: 22) subjects in each group compared to 41 (g: 13, s: 41) subjects required for pixel-wise phasor analysis with average standard deviation of (g: 0.0124, s: 0.0113). If cell-encircled analysis is feasible, then its use will better facilitate comparisons between groups with a smaller number of subjects for the detection of differences with age and disease.

The repeatability analyses indicated that phasor parameters have excellent consistency between measurements (<1% for g and 2% to 3% for s) but that the exponential fitting had moderate to poor consistency cell by cell. Interestingly, $\tau_{m}$ had higher repeatability than the other exponential-fit components in both the forward-backward scan analysis and the between-visit analysis. This is possibly because $\tau_{m}$ is the weighted average so individual variations between the parameters get averaged out. Nevertheless, the absolute value difference of exponential parameters across the forward-backward scans and between visits was about 5-10% for the lifetime parameters ($\tau_{m}$, $\tau_{1},$ and $\tau_{2}$) and ∼2% for $a_{1}$. Future work may need to investigate alternatives to the proprietary software used for exponential decay fitting in this work and methods to reduce variations between fittings. Additionally, the framewise repeatability has excellent consistency and lower absolute differences than the cell-by-cell analysis for both exponential and phasor parameters. Better consistency is potentially due to variability cell-to-cell that averages out resulting in better repeatability for averages across a location.

The eccentricity effect observed at 765/NIR and the age effect observed at 532/LSC with the lifetime and phasor components were similar to our previous work with the pixel-wise analysis and was attributed to changes in melanin concentration with eccentricity^34^ and increase in lipofuscin with age.^22^ Interestingly at 532/LSC, the change in phasor with eccentricity was clearer here than in the pixel-wise analyses.^47^ This is likely because there are fewer older subjects in the previous study and the advantage of analyzing low photon count data with the cell segmentation. Similarly, the cell segmentation analysis helped observe a trend toward a longer lifetime with age at 765/NIR, which was not shown in our previous study.^35^ With the 473/LSC, $\tau_{m}$ increased with eccentricity with the effect clearer than the pixel-wise approach. Although, the cause of the increasing lifetime with eccentricity was unclear. Future work will need to include characterizing more possible fluorophores, such as other constituents of lipofuscin to elucidate the source of eccentricity changes. Quantitative measurement of the change in lifetime with AOFLIO 473/SSC was possible for the first time with the cell segmentation approach; where $\tau_{m}$ had an average increase of 62.9 ps from the fovea to 12 deg, moving in the same direction as the clinical 473/SSC, which increased by ∼100  ps from the fovea to ∼15  deg.^63^ The magnitude may be different because clinical FLIO is measuring to a higher eccentricity and because it includes information from all of the retinal layers. The phasor location of the 473/SSC had no clear trend.

Previously, we postulated that the lutein/zeaxanthin ratio would be what is driving eccentricity changes in the 473/SSC channel;^64^ this is also what is suggested in clinical FLIO literature.^65^ Since the 473/SSC had no eccentricity effect, however, the fluorescence contributions may be more complex than previously thought with changes due to lutein and zeaxanthin but possibly also melanin^66^ and A2E.^67^ On the other hand, with increased axial sectioning, AOFLIO may exclude any fluorescence from the Henle nerve fiber layer (lutein and zeaxanthin) while focused on the RPE layer. Other fluorophores that may have short lifetimes that were not tested in the AOFLIO system are the protein-bound state of FAD^68^ and other constituents of lipofuscin. Future investigations with larger sample sizes or with specific retinal morphology, such as albinism needs to be conducted to further understand what is contributing to the 473/SSC signal.

The cell encircling analysis has utility in many situations. It allowed more locations in the AO 473/SSC to be analyzed with both exponential fitting and phasor analysis. In the 532/LSC, the pixel-based and cell-encircled analyses were well correlated, indicating that the cell encircling analysis may not provide any new information in photon-rich situations or when comparing the mean across an image. However, cell encircling is a very valuable tool for detecting changes between populations with a need for fewer subjects and potentially fewer photons. In cases where cellular visibility is able to be obtained with another modality and co-aligned to SWAF AOFLIO data, such as with NIRAF imaging, this analysis could allow an increased success rate of above-threshold areas, particularly in older or early disease subjects. In most of the AOSLO systems used to image the retina, NIR reflectance light is captured for location tracking and registration. Adding the detection channel to capture the NIR fluorescence intensity without increasing light exposure could potentially allow cellular-scale analysis in other photon-starved situations such as subjects with cataracts or reduced fixation and low autofluorescence.^69^ In diseased subjects where the cellular mosaic has been disrupted (such as druse or geographic atrophy), the tools developed here could be used to segment images into hyper- and hypo-fluorescent spots or into other regions of interest to investigate compositional changes across the transition zones at the cellular level. In addition, novel machine learning algorithms for segmenting cells can be developed to shorten the analysis time, increasing the relevance of this analysis. Co-capturing NIRAF intensity to encircle cells has the added bonus of reducing the photon count needed in the SWAF channels for lifetime analysis, which would reduce the patient’s exposure to the higher-energy, more uncomfortable light.

The difference between the spectral channels is not surprising and clearly visible in Fig. 11. Each channel is sensitive to different fluorophores and receives relatively different intensities from each fluorophore ($a_{1}$), which both impact the mean lifetime. The autofluorescence in the RPE arising from lipofuscin, melanin and melanolipofuscin can possibly be differentiable by various spectral channels. Lipofuscin can be primarily observed in the 532/LSC and 473/LSC while melanin has a strong presence in the 765/NIR channel. Melanolipofuscin can potentially be observed in both channels. The 532/LSC avoids exciting lutein, zeaxanthin, and elastin, primarily receiving signal from lipofuscin with some effects from FAD, all-trans-retinal, and melanin.^24^^,^^47^ Additionally, 532 nm excitation avoids the macular pigment absorption, improving signal at the fovea, as compared to excitation at 473 nm.^51^^,^^52^ A combination of the 532/LSC and 765/NIR channels should give a comprehensive view of the melanin, melanolipofuscin, and lipofuscin autofluorescence in RPE.

The 765/NIR channel receives autofluorescence primarily from melanin and melanin-related products.^70^ This is confirmed by the eccentricity trend of a decreasing $a_{1}$ (Fig. 4), which agrees with optical density of melanin decreasing with eccentricity.^19^^,^^71^^,^^72^ Melanin can have density-dependent self-absorbance of its fluorescence emission, which causes self-quenching by secondary absorbance. Since this is the case, microenvironment changes may occur, which could account for the variations observed in the 765/NIR channel.^73^ In the 532/LSC, however, there is not a clear variation with eccentricity (Fig. 6) but there is a clear lengthening of lifetime with age (Fig. 7), which is possibly due to an increase in melanolipofuscin with age, moving the lifetime longer. Note the decrease of $a_{1}$ with age, which may be indicating an increased effect of melanolipofuscin with age and a reduction of lipofuscin contribution to the signal. Because the 765/NIR channel has a strong eccentricity effect and the 532/LSC has a strong age effect, multispectral imaging with these two wavelengths may be a powerful tool to non-invasively investigate changes in composition and function of the RPE cells with disease.

This work investigates multispectral cell-to-cell changes of fluorescence lifetime with eccentricity and qualitatively with age. The lack of an eccentricity effect from the AOFLIO 473/SSC indicates that multiple fluorescent sources, which change with eccentricity are likely contributing to the signal including but not limited to lutein, zeaxanthin, A2E, FAD, and melanin. The ability to combine pixels from a single cell into one histogram allows for the fluorescence lifetime to be analyzed in scenarios with low-photon counts. Specifically, the 765/NIR channel is recommended to be imaged in parallel to any desired visible channel of interest and co-aligned to achieve cellular analysis at any wavelength, because excitation in the NIR has an increased rate of cellular visibility^19^ and is more comfortable for the eye.^74^ With the parallel imaging of the 765/NIR channel, light exposure to high-energy SWAF channels can be reduced due to feasibility of data analysis with lower photons; patient comfort could be improved with lower light requirements; subjects with optical opacities, such as cataracts, strong photophobia, reduced fixation quality, such as children or those with low visual acuity, and patients with reduced autofluorescence intensity may now be able to be imaged. Capture of both the 532/LSC and 765/NIR channels has the potential to quantify changes in fluorescence with age and eccentricity specifically due to variations in lipofuscin, melanin, and melanolipofuscin, which may prove to be a potentially useful label-free method for tracking diseases. This paper represents the first multispectral, cellular-scale fluorescence lifetime comparison of the *in vivo* human RPE mosaic.

## Supplementary Material



## Data Availability

All data in support of the findings of this paper are available within the article or as Supplementary Material. The individual lifetime data utilized in this study are available from the authors upon request.
