# Supplementary material for: Multispectral label-free in vivo cellular imaging of human retinal pigment epithelium using adaptive optics fluorescence lifetime ophthalmoscopy improves feasibility for low emission analysis and increases sensitivity for detecting changes with age and eccentricity
Source: J Biomed Opt. 2024 Jul 3;29(Suppl 2):S22707. doi: 10.1117/1.JBO.29.S2.S22707 (PMC11221116; doi:10.1117/1.JBO.29.S2.S22707)
Supplement: Supplementary file 1 [file JBO_029_S22707_SD001.docx]

Multispectral label-free *in vivo* cellular imaging of human retinal pigment epithelium using adaptive optics fluorescence lifetime ophthalmoscopy improves feasibility for low emission analysis and increases sensitivity for detecting changes with age and eccentricity

Karteek Kunala,^a,*,†^ Janet A.H. Tang^†^,^b,c^ Keith Parkins,^b^ Jennifer J. Hunter ^b,c,d^

a Byers Eye Institute, Stanford University, Palo Alto, CA 94303, USA

b Center for Visual Science, University of Rochester, Rochester, NY 14627, USA

c The Institute of Optics, University of Rochester, Rochester, NY 14627, USA

d School of Optometry and Vision Science, University of Waterloo, Waterloo, ON N2L 3G1, Canada

†**co-author (equal contribution)**

*Address all correspondence to Karteek Kunala, kkunala@stanford.edu


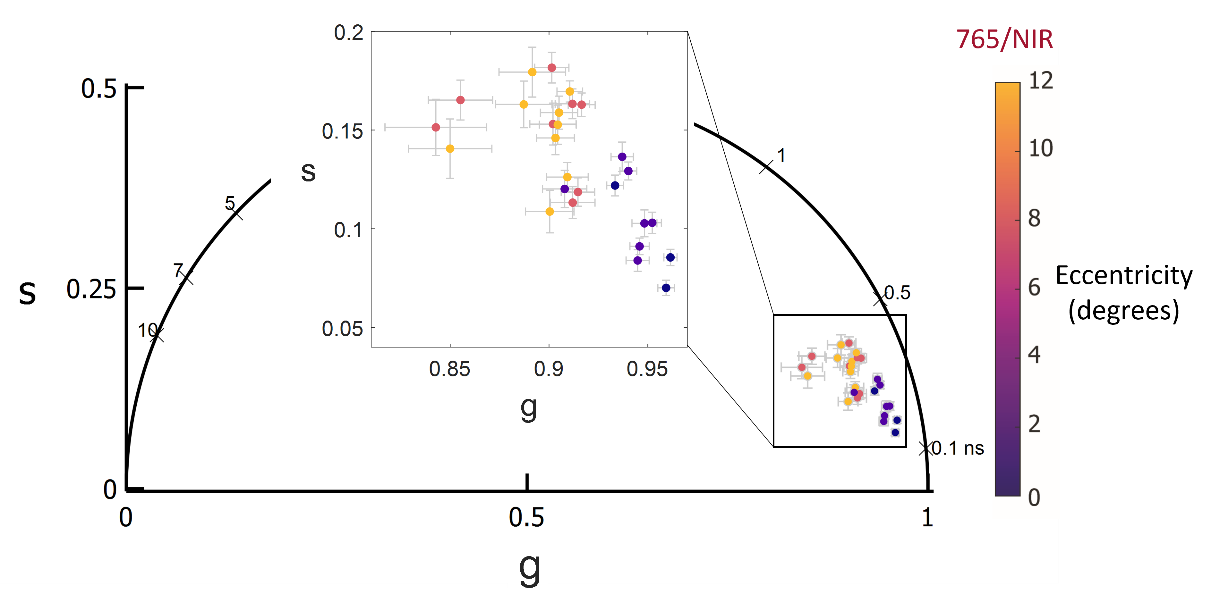


Fig. S1 Phasor location moves up and to the left towards longer lifetimes with eccentricity in the 765/NIR channel.


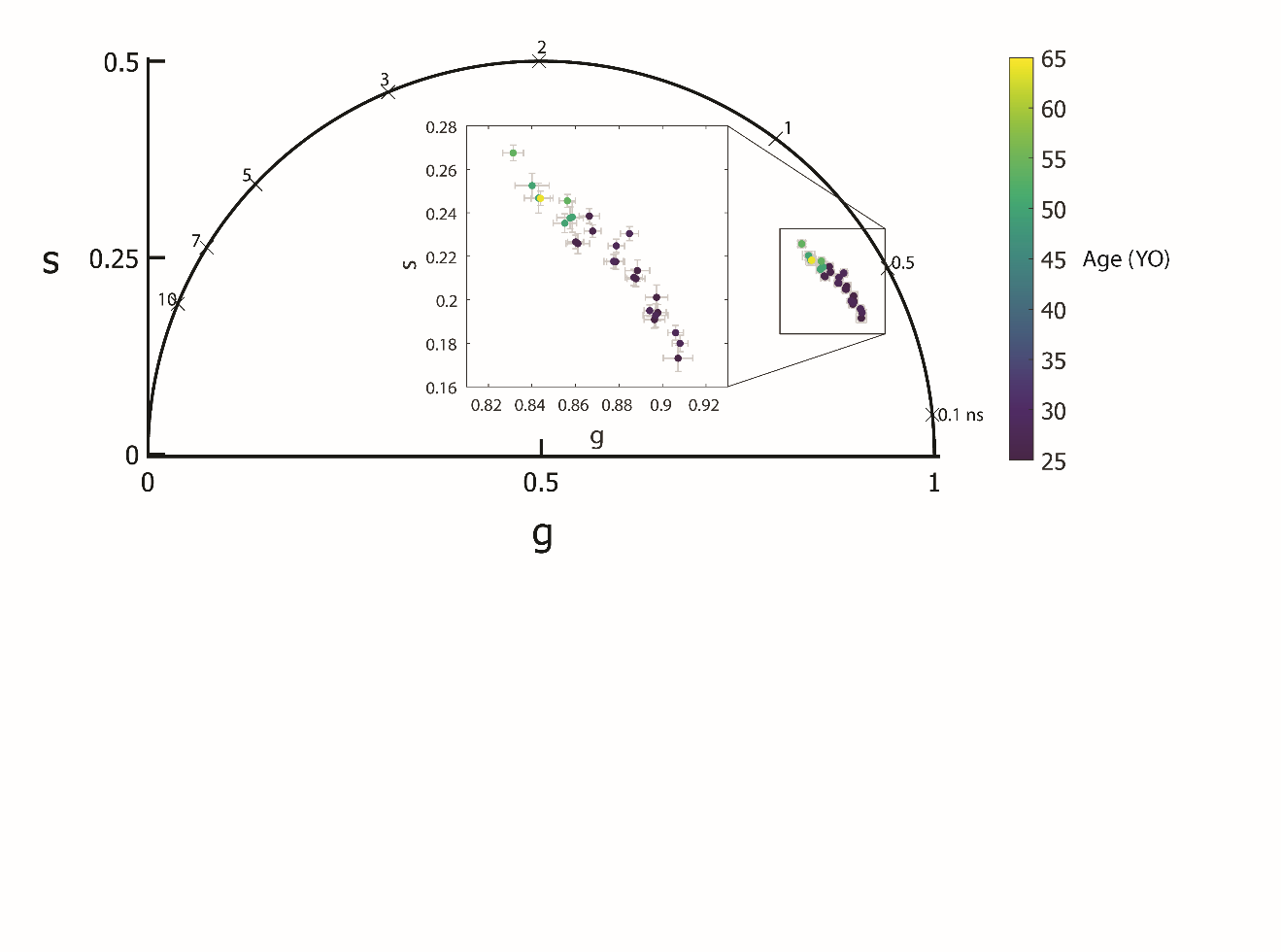


Fig. S2 Phasor location moves up and to the left towards longer lifetimes with age in the 532/LSC.


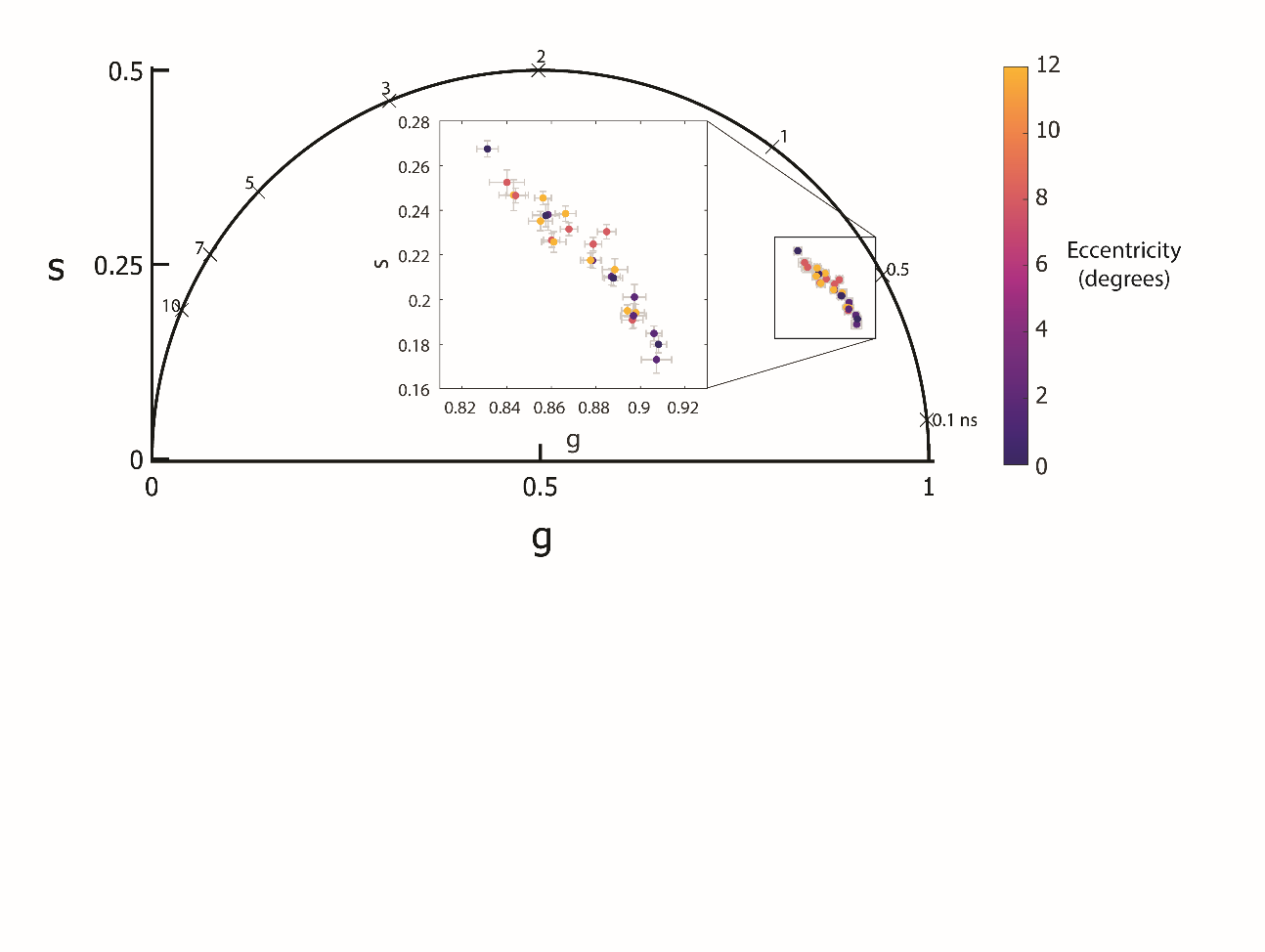


Fig. S3 Phasor location trends to the left towards longer lifetimes with eccentricity in the 532/LSC.


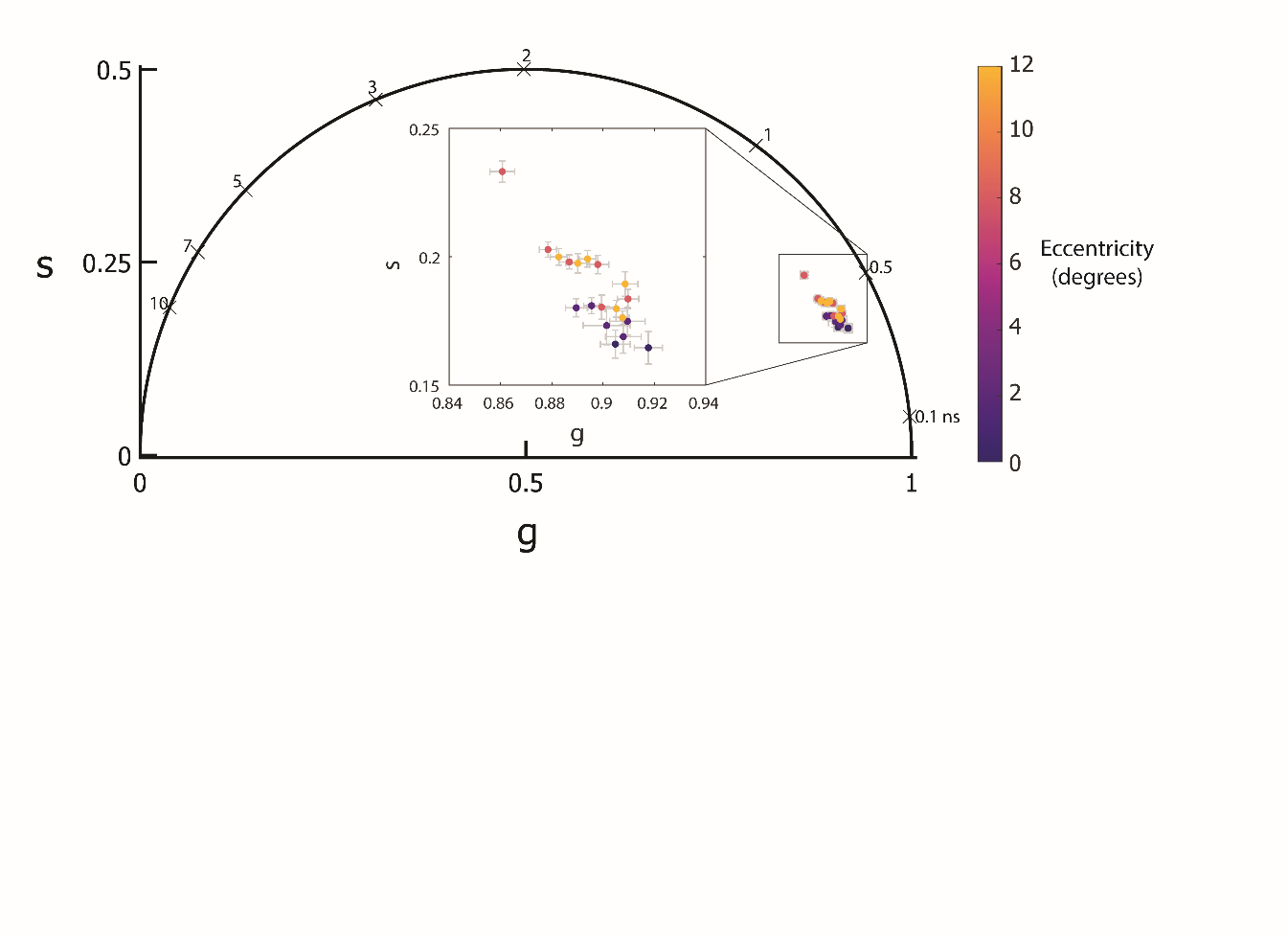


Fig. S4 Phasor location moves up and to the left towards longer lifetimes with eccentricity in the 473/LSC similar to the 532/LSC.
